# Effect of Visual Acuity on the Surgical Outcomes of Secondary Sensory Strabismus

**DOI:** 10.4274/tjo.67878

**Published:** 2015-12-05

**Authors:** Kadriye Erkan Turan, Hande Taylan Şekeroğlu, Emin Cumhur Şener, Ali Şefik Sanaç

**Affiliations:** 1 Hacettepe University Faculty of Medicine, Department of Ophthalmology, Ankara, Turkey; 2 Private Practice, Ankara, Turkey

**Keywords:** Secondary, sensory, Strabismus, visual acuity

## Abstract

**Objectives:**

To investigate the outcomes of secondary sensory strabismus surgery and to discuss the effect of visual acuity on success.

**Materials and Methods:**

The medical records of patients with sensory strabismus who underwent recession-resection on the eye with vision loss were reviewed. Only patients with visual acuity of ≤0.2 in the operated eye were enrolled. Data including age at surgery, visual acuity, etiology of vision loss, preoperative and postoperative deviations, follow-up duration, and surgical outcomes were recorded. Success was defined as a final deviation of ≤10 prism diopters (PD). To evaluate the effect of visual acuity on postoperative success, patients were grouped as follows according to the visual acuity of the operated eye: group 1, visual acuity <0.05; group 2, 0.05-0.1; and group 3, 0.125-0.2.

**Results:**

Ten females and 14 males met the inclusion criteria. The mean age at surgery was 21 years (range, 6 to 56 years). The mean preoperative deviation angle was 52.7 PD (range, 20 to 80 PD). Age at surgery, preoperative deviation and follow-up time were similar in patients with esotropia (n=7) and exotropia (n=17) (p>0.05 for all). The success rate was 62.5% at short-term and 42.1% at long-term follow-up. There was no statistically significant difference in short-term success rate among visual acuity subgroups (p=0.331), whereas the difference was statistically significant at long-term follow-up (p=0.002). The long-term success rate was higher in group 3 compared to groups 1 and 2.

**Conclusion:**

Better visual acuity seems to be a potential predictor for higher long-term success after strabismus surgery in patients with sensory strabismus.

## INTRODUCTION

Sensory strabismus is generally defined as secondary deviation resulting from unilateral vision loss caused by anisometropic amblyopia or organic pathologies such as optic nerve or retinal abnormalities, corneal opacity and cataract. Sensory deviations emerge from an underlying sensory deficit followed by partial or complete disruption of fusion.^1^

Surgery for sensory strabismus should be considered as a reconstructive procedure due to the negative psychosocial impact of sensory strabismus on patients.^2,3^ A recession-resection operation of the eye with vision loss or lower visual acuity is the most commonly preferred surgery. Because the improvement of binocular function is mandatory for higher success in strabismus surgery, long-term maintenance of ocular alignment is difficult after surgical correction of sensory strabismus due to its unique nature.^4^

The purpose of this study was to analyze the short- and long-term outcomes of strabismus surgery for horizontal secondary sensory strabismus and to emphasize the effect of visual acuity on surgical success.

## MATERIALS AND METHODS

The clinical records of patients with horizontal sensory deviations who underwent strabismus surgery between 2004 and 2013 were reviewed upon approval of the institutional ethics committee. The authors adhered to the tenets of the Declaration of Helsinki. Patients with monocular vision loss due to anisometropic amblyopia or organic pathologies with at least 2 months postoperative follow-up were enrolled in the study. Visual acuity was measured by Snellen chart and only patients with a visual acuity of 0.2 or lower in the operated eye were included. Exclusion criteria were as follows: bilateral visual impairment, vision loss due to strabismic amblyopia, paralytic and restrictive strabismus, previous ocular surgery, previous botulinum toxin injection and coexisting vertical deviation. Patients who underwent adjustable suture surgery were also excluded.

The informed consent comprised information concerning alternative approaches including botulinum toxin injection, adjustable sutures, bilateral surgery, and possible outcomes, particularly limitation in horizontal gaze positions, enophthalmos, and narrowing of the lid fissure. Only patients who opted for the unilateral recession-resection procedure confined to the eye with vision loss were included. Seven patients with esotropia and 17 patients with exotropia met the criteria.

Detailed medical history, age at surgery, gender, etiology of the vision loss, visual acuity, type of deviation, and anterior segment and fundus examination findings were recorded. The angle of preoperative and postoperative deviations were assessed by Hirschberg test, Krimsky test or alternate prism cover test (in prism diopters [PD]) when possible. Both near and distance measurements were taken when possible. The average of the near and distance deviations were used for surgical planning. As the main purpose was to evaluate the effect of visual acuity on postoperative success, the patients were classified according to the visual acuity of the operated eye as follows: group 1, visual acuity <0.05; group 2, 0.05-0.1; or group 3, 0.125-0.2. Sensory fusion was evaluated with Worth 4-Dot test and stereopsis was assessed with Titmus test.

All patients gave informed consent for unilateral recession and resection surgery. All operations were performed using limbal incisions. The postoperative surgical target was orthophoria in primary position.

A successful outcome was defined as esodeviation or exodeviation of 10 PD or less in primary position. The final outcome was assessed in the short and long term. The first postoperative visit was evaluated as short-term (2-5 months), whereas the final visit was considered long-term follow-up (9-77 months). All patients had short-term follow-up and 19 patients also had long-term follow-up.

Statistical analysis of the results was performed with SPSS version 15.0 (Statistical Products and Service Solutions, SPSS Inc., Chicago, IL). Mann-Whitney U test was used to compare numerical data; chi-square and Fisher’s exact test were applied to compare the success rates. Differences among groups were analyzed using Kruskal-Wallis tests. Conover’s multiple comparison test was used as a post hoc test. The Friedman test was used to compare the angle of preoperative and postoperative deviations. Descriptive statistics were expressed as mean ± standard deviation (min-max). Qualitative data are summarized using frequency and percentages. Level of significance was accepted as α=0.05.

## RESULTS

Twenty-four patients (10 females and 14 males) met the criteria for inclusion among the patients who underwent surgery for sensory strabismus between 2004 and 2013. Of the 24 patients, 7 (29.2%) had esotropia and 17 (70.8%) had exotropia. The mean age at surgery was 21 years (range, 6 to 56 years). The mean preoperative deviation angle was 52.7 PD (range, 20 to 80 PD). The mean short-term follow-up time was 2.79 months (range, 2 to 5 months) and the mean long-term follow-up time was 26.3 months (range, 9 to 77 months). There were no significant differences in age at surgery, preoperative deviation, or short- and long-term follow-up time between the esotropia and exotropia groups (p>0.05 for all).

Structural abnormalities (previous perforating injury, corneal opacity, choroidal coloboma, retinopathy of prematurity, toxoplasma chorioretinitis) were the most common causes of vision loss and were found in 10 (41.7%) patients. Six (25%) patients had anisometropia, 5 (20.8%) patients had optic nerve pathologies (optic atrophy, optic disc coloboma and morning glory anomaly) and 3 (12.5%) had unilateral congenital cataract. The causes of vision loss were congenital in 15 (62.5%) patients (6/7 esotropia, 9/17 exotropia) and acquired in 9 (37.5%) patients (1/7 esotropia, 8/17 exotropia).

Visual acuity ranged from no light perception to counting fingers in 9 patients (group 1), from 0.05 to 0.1 in 8 patients (group 2), and from 0.125 to 0.2 in 7 patients (group 3). There was no significant difference in horizontal deviation type among visual acuity groups (p=0.857). The mean age at surgery was significantly younger in group 3 compared to group 1 and group 2 (p=0.006). There were no significant differences in preoperative deviation or short- and long-term follow-up times among visual acuity groups (p>0.05 for all). The findings are summarized in Table 1. None of the patients had fusion and stereopsis preoperatively or postoperatively.

The amount of resection and recession are given in detail in Table 2. There were no surgery-related complications.

The mean postoperative deviation was 15.3 PD (range, 0 to 45 PD) at the short-term follow-up. The mean horizontal deviation was significantly decreased after surgery (p<0.001). None of the patients were overcorrected. Six patients in the esotropia group and 13 patients in the exotropia group had long-term follow-up, at which the mean postoperative deviation angle was 20.5 PD (range, 0 to 52 PD). The overall success rate after strabismus surgery was 62.5% at short-term and 42.1% at long-term follow-up. There were no statistically significant differences in postoperative deviation or success rate among both groups at short- and long-term follow-up (Table 3). Twelve (50%) patients had slight limitations in extreme horizontal gaze positions. Limitations were not related to surgical dosages. None of the patients complained about limitation of horizontal gaze, enophthalmos or narrowing of lid fissure and none of them experienced diplopia postoperatively.

There was no statistically significant difference in short-term success rate among the three visual acuity subgroups (p=0.331), whereas the difference was statistically significant at long-term follow-up (p=0.002). The long-term success rate was higher in group 3 in comparison to groups 1 and 2. The success rates of esotropic and exotropic patients in visual acuity subgroups are given in Table 4. When the effect of visual acuity on long-term surgical success was further analyzed for eso- and exotropic patients separately, it was found that there was no significant relation between visual acuity and success rate in esotropic patients; however, success rate was significantly related to visual acuity in exotropic patients (p=0.007).

Gender, age, etiology of vision loss, preoperative deviation angle, and surgical dosage were found to have no significant influence on surgical success rates in the esotropia and exotropia groups (p>0.05 for all).

## DISCUSSION

The clinical factors that affect the success of strabismus surgery have been reported as visual acuity, preoperative deviation angle, axial length, refractive error, age of onset, age at surgery, surgical technique, surgical amount, stereopsis, and fusion.^5,6,7,8,9,10^ Since the underlying problem is the alteration of binocular function that should actually be present preoperatively or improved postoperatively for successful surgical outcome, it can be presumed that the surgical success would be lower in cases with sensory strabismus. With this study, we were able to demonstrate that visual acuity may predict the long-term maintenance of ocular alignment in sensory strabismus.

In sensory strabismus, surgery on only the eye with poor vision is generally preferred because it may be difficult to persuade the patient to undergo a surgery on the healthy eye.^4^ In addition, long-term maintenance of ocular alignment in sensory strabismus is expected to be poor, and recurrence is high due to the low possibility of fusion gain.^11,12^ However, surgery should not be considered only an aesthetic procedure, but also a reconstructive surgery. In addition to addressing the negative psychosocial impact of the condition on patients, the possible functional improvements should not be underestimated.^2,3^ Some authors reported that the botulinum toxin is a good alternative to surgery for this type of strabismus, as it is a noninvasive technique.^13,14,15^

There are few published studies concerning the surgical results of sensory strabismus. Oral et al.^16^ reported a 75.9% surgical success rate overall, with 87.5% for the exotropia group and 61.5% for the esotropia group. Oliveira et al.^17^ reported a 90% surgical success rate, most with adjustable sutures, and success was defined as a residual deviation of up to 15 PD. Yurdakul^18^ found acceptable outcomes in 73.9% of esotropia cases and 80.6% of exotropia cases with one surgery after a follow-up period of at least one year. Merino et al.^19^ also reported a high surgical success rate in sensory strabismus in patients who underwent unilateral, bilateral or multiple procedures. On the other hand, Portes et al.^20^ found a surgical success rate of approximately 50% in exotropic patients with at least 6 months of follow-up. Martinez et al.^21^ reported a 41.2% surgical success rate for amblyopic patients with esotropia. In the present study, 15 (62.5%) patients had postoperative deviation within 10 PD at short-term follow-up with a mean of 2.79±1.06 months. Of the 19 patients who were followed up for more than 9 months postoperatively, 8 (42.1%) showed alignment within 10 PD at the last follow-up with a mean of 26.26±17.4 months.

Havertape et al.^22^ reported that esotropia was more common in patients suffering from a congenital loss of vision, and exotropia was more prevalent in patients whose vision loss was acquired. Oral et al.^16^ suggested that success might be more limited in sensory esotropia due to congenital causes in particular. In the present study, the causes of vision loss were congenital in 15 (62.5%) patients (6/7 esotropia, 9/17 exotropia) and acquired in 9 (37.5%) patients (1/7 esotropia, 8/17 exotropia). However, contrary to the literature, surgical success rate was lower in our exotropic patients. Furthermore, a correlation between long-term success and visual acuity level was found for exotropia. The significant difference in age is another point that may draw attention. It is noteworthy that the mean age of patients with higher visual acuity (group 3) was significantly lower compared to the other two groups. This difference may explain the higher long-term success rate in group 3, as younger patients may have a higher chance to obtain a certain amount of sensory fusion in time which could not be demonstrated in the present study or, in other words, surgery in childhood might have improved success.

In all cases, surgery was performed only on the eye with vision loss. If the deviation angle is too large to be corrected, the surgeon faces the challenge of deciding between additional one- or two-muscle surgery on the healthy eye or an extra-large amount of surgery on the eye with vision loss alone. If the surgeon plans unilateral surgery, limitation in horizontal gaze positions should be also kept in mind.^23^ This possibility was discussed in detail prior to surgery with the patients in our study and all surgical options were proposed. They all chose unilateral extra-large recession-resection procedure. None of the patients reported diplopia or cosmetic concerns, even though 12 patients had limited movements in horizontal positions of gaze.

In the present study, a correlation between long-term success and the visual acuity of the operated eye was observed for exotropia. Gusek-Schneider and Boss^24^ showed a correlation between the postoperative angle for far distance and the visual acuity for secondary divergent strabismus.

The results of the present study should be viewed in the context of some limitations. First, the number of patients was small and was not equally distributed between the esotropia and exotropia groups. The retrospective design of the study added its own limitations as well. Factors other than visual acuity such as fixation quality and characteristics which may contribute to the surgical success were considered beyond the scope of this study and therefore not analyzed, but may be the main topic of further prospective studies. Finally, it should be borne in mind that the results of the present study cannot be extrapolated to all patients with sensory strabismus.

In conclusion, better visual acuity may predict better outcome in terms of long-term success in patients with sensory strabismus. However, larger prospective randomized studies are still needed to analyze and ascertain the clinical factors that contribute to the surgical outcomes.

## Figures and Tables

**Table 1:**
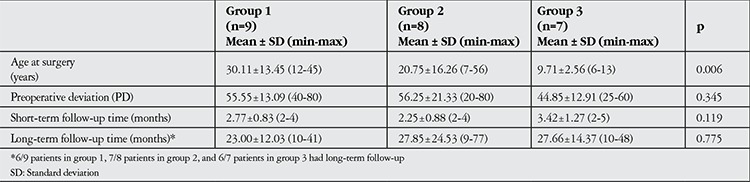
The comparison of age at surgery, preoperative deviation and follow-up times of visual acuity subgroups (Kruskal-Wallis test)

**Table 2:**
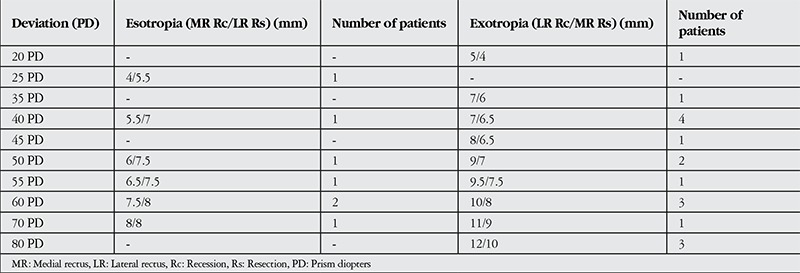
Individual surgical dosage for eso- and exodeviations

**Table 3:**
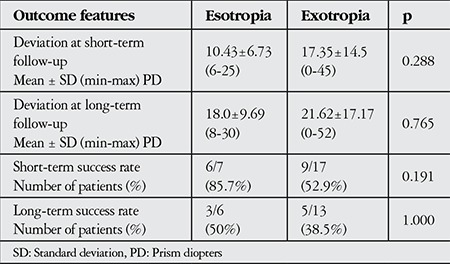
The postoperative clinical features at short- and long-term follow-up (Mann-Whitney U test and Fisher’s Exact test)

**Table 4:**
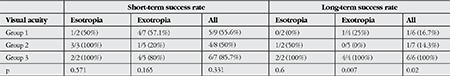
The comparison of short- and long-term success rates of esotropic and exotropic patients according to visual acuity subgroups
